# Promoting nutrition literacy in children: a case study of a community partnership between a university and an elementary school

**DOI:** 10.5195/jmla.2024.1678

**Published:** 2024-04-01

**Authors:** Candise Branum

**Affiliations:** 1 branum@gonzaga.edu, Assistant Professor & Health Sciences Librarian, Gonzaga University, Spokane, WA

**Keywords:** Academic libraries, Community Engagement, Community Outreach, Health Information Literacy, Nutrition, children's health

## Abstract

**Background:**

Health literacy outreach is commonplace within public and hospital libraries but less so in academic libraries, where it is often viewed as not integral. Academic health science libraries may collaborate with public libraries to provide public health information literacy programming or “train the trainer” sessions, but examples of academic health science librarians leading community health initiatives are still limited.

**Case Presentation:**

This case report discusses a collaborative project between Gonzaga's Foley Center Library, the School of Nursing and Human Physiology, and a local elementary school to promote health literacy for students and their families, led by an Academic Health Sciences Librarian. The project scope included delivering nutrition education to elementary school students and their families, but pandemic closures limited plans for in-person programming. Conversations with stakeholders led to additional project opportunities, including tabling at the local block party, collaborating on a campus visit for 5th and 6th graders, supporting middle school cooking classes, and the creation of a toolkit for elementary and middle school teachers to support curriculum about healthy body image and potential disordered eating.

**Conclusion:**

This project demonstrates one example of how academic libraries can partner with other campus departments to support health literacy outreach in their local communities. The pandemic made planning for in-person programming tenuous, but by expanding meetings to include staff from other areas of the university, the project team was able to tap into additional outreach opportunities. This work fostered close relationships with the local elementary school, providing the groundwork for collaborative health programming in the future, though more thorough assessment is suggested for future projects.

## BACKGROUND

Healthy People 2030 defines personal health literacy as, “the degree to which individuals have the ability to find, understand, and use information and services to inform health-related decisions and actions for themselves and others” [1]. Libraries have been involved in health literacy collaborations for decades; much of this work has been supported by the National Library of Medicine, which has been a leader in providing funding for health literacy outreach programming [2]. In Duhon and Jameson's 2013 survey, only 28% of general academic libraries supporting four-year institutions participated in health information outreach, with 61% reporting little or no need for health information outreach; although budgetary and staffing limitations were listed as reasons for not participating in health outreach, some felt that health outreach was not a part of the institution or departmental mission [3].

In a 2016 literature review of library participation in health literacy programming, the vast majority of studies reviewed were conducted by public or hospital libraries; in cases where academic health science librarians were involved, programming was primarily done in collaboration with public library partners [4]. Health science librarians have advanced knowledge of finding and evaluating health resources and experience in developing curriculum, while public librarians are able to communicate effectively with diverse communities that have varying levels of information literacy, and combining these skillsets and resources provides the opportunity to serve a larger sector of the community [5]. Partnerships with public libraries allow academic health science librarians to utilize their expertise by providing health literacy programming to the general public; in particular, many workshops have been geared towards providing seniors with the basic health literacy resources [6–9]. Examples of innovative programs involving academic health science librarians and public libraries include inviting medical school staff and students [10] or library science students [11] to deliver public health literacy programming.; in particular, many workshops have been geared towards providing seniors with the basic health literacy resources [6–9]. Examples of innovative programs involving academic health science librarians and public libraries include inviting medical school staff and students [10] or library science students [11] to deliver public health literacy programming.

The involvement of academic libraries in community health literacy often takes the form of a “train the trainer” program, where health sciences librarians provide training to public librarians or community members who may interact with health questions [11–17]. Additionally, academic health science librarians can be involved in health literacy through the creation of online research guides and toolkits; this includes consumer health websites that support the local community [11,18,19], as well as health literacy resources for healthcare professionals [11]. This case study describes a grant funded project to enhance health literacy within a local community, managed by an academic health science librarian at a university not affiliated with a medical school or hospital.

The primary goal of this project was to promote community health partnerships, with health promotion as the core function. The bulk of this work was conducted in the form of nutrition literacy sessions with elementary school students, and while learning outcomes were developed for each class, student learning was not measured. Outside of the classroom, success was measured through the ability to distribute nutrition education information to community members; the scope of this project focused on promotion of information, which could be seen through point of contact with the community and in particular, the handing out of print materials.

A secondary outcome of this project was to further situate Gonzaga University as a source of support for health education projects. Gonzaga's Center for Community Engagement has previously collaborated on many health and wellness programs within the local Northeast Spokane community, including hosting a health resources fair for residents at Gonzaga Family Haven (a housing project for previously unhoused Spokane residents) and funding a part-time mental health counselor at the local Elementary School. While health partnerships are not uncommon at Gonzaga, this program was the first to involve the Foley Library as educational partners and to provide classroom programming in collaboration with teachers at Logan Elementary. Additionally, this project specifically focused on classroom teaching, and helped further develop the University as an educational partner in health literacy programming.

This project also allowed the author, a Health Sciences Librarian who was brand new to the institution, the opportunity to build relationships with administrators in the School of Nursing and Human Physiology, positioning them as a potential collaborator in future health literacy projects. As a white academic, the author was concerned about the power structures and privilege embedded in community health outreach initiatives, which could potentially impact relationships with external partners. To mitigate this, the author viewed their role not as an expert instructing the direction of the project, but as a facilitator, consulting Logan Elementary teachers about specific needs and gaps that could be addressed throughout the grant period. The author hopes that this case study can help other librarians navigate similar situations in their own institutions.

## CASE PRESENTATION

Gonzaga University is a private Jesuit liberal arts institution; while the School of Nursing and Human Physiology (SNHP) provides undergraduate, graduate, and doctoral degrees, the University is not affiliated with a medical school or hospital and the Foley Library supports graduate and undergraduate programs across a variety of disciplines. Gonzaga University is a Jesuit institution with a strong commitment to social justice and community enrichment, but also resides on stolen land and was built on the broken promise of providing education for Native American students [20]. With this legacy in mind, it was important to build programming that did not situate the project team (white faculty and staff at an private academic institution) as intellectual heroes, but instead allowed for nuanced conversations in partnership with the elementary school educators who best understand the needs of their students. Rather than building completely new and innovative programming, the goal was to work alongside the teachers to supplement the health literacy work that was already taking place.

Gonzaga University resides in the Logan neighborhood in Spokane, Washington, where 25% of residents live below the poverty level; food insecurity in children has increased alongside the COVID-19 pandemic, and access to healthy food continues to be a concern [21]. The Washington Food Security Survey found that during the pandemic, the number of households in the Logan neighborhood that use food assistance programs rose from 32% to 41%, with 40% of respondents saying their diets had worsened during the pandemic [22]. Gonzaga supports multiple programs to address food insecurity in the Logan neighborhood, including partnerships with Spokane Public Schools, Second Harvest Food Bank, Sodexo food services and the University's Campus Kitchen. In conversations between Elementary school administrators and Gonzaga's Center for Community Engagement about how Gonzaga can help support students, it was recognized that in addition to access to healthy food, there was also a need for education around nutrition literacy, and administrators requested that the University provide support for health literacy education with students and their families.

### Description of the Project

In response to this request for health literacy support by school administrators, leadership in the SNHP and the Foley Center Library collaborated on applying for American Rescue Plan Act (ARPA) grant funding through the Institute of Museum and Library Services (IMLS) to support this work. Over a six month period, this grant provided $9,960 in funding, which was used to purchase materials to support in-person educational programming, print materials used for outreach, pre-packaged healthy food for distribution at community events, and the hiring of a graduate research assistant. The author of this case study, a Health Sciences Librarian at Gonzaga's Foley Library, was not involved in the writing of the grant application, as they were not employed by the institution during these early planning stages. Instead, they inherited management of the program upon their arrival at Gonzaga in late 2021.

This collaborative project focused on increasing the ability of students and families to understand health information, potentially leading to more informed health decision making. Health literacy is a broad topic, but the group chose to initiate this work through basic nutrition education. While students were the primary target audience for health literacy programming, school officials noted that health literacy education should extend to student families as well, as children generally do not have control over meal planning or health decisions.

The project team, which consisted of the author, the Assistant Dean for the SNHP, and the SNHP Program Manager, met bi-monthly with members of Gonzaga's Center for Community Engagement (CCE) and Logan Elementary School administrators and teachers. Approximately 6-10 people were in attendance at these regular meetings, where the author (as the project lead) provided updates and sought out feedback from community partners. The CCE has partnered with Logan Elementary on a number of other programs, and these regular check-in meetings provided the opportunity for program leads to share updates, get feedback, and coordinate schedules. Through these conversations, two primary venues were identified by our community partners for conducting this work: Educational programing with children at Logan Elementary and community outreach at the Logan Family Dinners.

### Elementary School Programming

The first major component of the grant was to provide nutritional literacy programming to students at the local elementary school, to be developed and delivered by the author. In 2021 The author and the project team began meeting regularly with administrators and the physical education and health teacher from Logan Elementary to discuss the development of this program.

Numeracy literacy was initially listed as a program objective, specifically in teaching students how to read and understand nutrition labels. Upon joining the team and reading the project description, the author was deeply uncomfortable with introducing concepts that could easily slide into supporting disordered eating behaviors [23] or unintentionally shaming children around food choices (of which they generally had no control over). Through conversations with school administrators, the author had numeracy literacy removed as an objective and the group decided to focus specifically on nutrition literacy. Following suggestions for promoting healthy food, the team centered programming on the joys and positive benefits of foods rather than applying moral judgment to foods [24].

The author created a tiered curriculum for K-6 students, much of it modified from the USDA's MyPlate curriculum [25]. Lesson plans from MyPlate curriculum were modified to meet the needs of each grade level, and student understanding was assessed by reviewing completed assignments. Health Education Curriculum Analysis Tool (HECAT) was used to help identify learning outcomes for each grade level [27]; Table 1 describes each learning outcome and the associated lesson plan for each level.

**Table 1:** Summary of K-6 Learning Outcomes and Activities

GRADE LEVEL	LEARNING OUTCOMES	ACTIVITIES
Kindergarten	Explain the importance of trying new foods Identify some nutritional benefits of eating fruits	Storytime: *I Will Never Not Ever Eat a Tomato*. Students walk to different stations to try fruits (each of a different color) and learn one nutritional fact about what that fruit does for their body (i.e., helps with vision, provides energy, etc.).
1st - 2nd	Explain the importance of choosing nutrient-dense foods and beverages that help people feel good. Explain the importance of eating different foods from all the food groups Demonstrate ability to place foods in one of the five food groups	Dance and sing along to “Alive with Five!” song. Students complete a worksheet describing their favorite food, what other food they might eat it with, and what food group it is in. Students then draw a picture of their food choice.
3rd - 4th	Describe the benefits of eating plenty of fruits and vegetables. Describe how to make or choose good-tasting, nutrient dense snacks.	Provide a short presentation about how vitamins and minerals affect their body. In small groups, students create a recipe for a snack fit for an athlete; write out the recipe, describe why they chose it and what it can do for their bodies. Groups then create a poster advertising their product and share it with the class.

Health literacy programming for K-4 classes took place at Logan Elementary. The project team planned to host the 5th and 6th grades in Gonzaga's Foley Library for the health literacy programming. When the team learned that the CCE staff was also planning on hosting the 5th and 6th grades on campus in a similar time frame, the two teams collaborated in planning the campus visit.

While pandemic infections did affect student attendance at the start of the winter term, as the year progressed, rates of attendance began to return to near-normal levels, and in-person programming was delivered to approximately 260 elementary school students. The team collected basic data on the student health literacy sessions by reviewing student work and collecting feedback from elementary school teachers who participated in the program. Some general indicators of the positive impact of the program were found in this data. For example, almost all the first and second grade students were able to correctly identify the food groups for their favorite foods. Another example was that all grade 3 and 4 students successfully completed the assignment that included creating a recipe fit for an athlete, making a poster advertising their product, and explaining how the vitamins or minerals found in each ingredient could contribute to increasing athletic performance.

Halfway through the six-month grant period, the school principal asked for assistance in creating an intervention for 5th and 6th grade students to combat potential disordered eating; as no one on our team was qualified to teach this topic, we were unable to provide an intervention. However, the team was able to incorporate a facilitated discussion about body image and student attitudes about food to learn more about student feelings. Through these conversations, a small group of students was identified as having negative feelings about food, directly stating that they do not eat lunch, though this was not the majority of students in the cohort. The team also found that a group of students cooked for themselves, including doing most cooking for themselves, but because they didn't know how to cook, they ended up mostly eating “unhealthy foods.”

The author also built a toolkit to support local teachers looking to introduce workshops or lesson planning around addressing disordered eating. Built on the LibGuides platform, this toolkit was shared with administrators at Logan Elementary, as well as Shaw and Yasuhara Middle Schools, and received nearly 300 visits in the first four months of being published.

### Community Outreach

The second major component of this grant project was community outreach through a preexisting program supported by Gonzaga, the Logan Family Dinners. The program provides free weekly dinners at Logan Elementary and acts as a community gathering space that has historically been well-attended. Partnering with an established program allowed for a connection between health literacy education and the specific meals being offered. When COVID-19 infection rates began to spike in January of 2022, the Logan Family Dinners canceled all plans for in-person dining and instead provided to-go dinners. The project team was disappointed that they would be unable to connect with people in-person. However, the event organizers noted that people who attended the dinners were very receptive to print materials in the past and suggested that handouts be created to be distributed with to-go dinners. The project graduate research assistant, who was pursuing a degree in Clinical Mental Health Counseling, created five separate handouts geared towards parents on topics such as intuitive eating, nutritional guidelines, and disordered eating in adolescents, while the author designed placemats for students that included information about nutrition and healthy bodies (see Table 2).

**Table 2:** Summary of All Program Activities

ACTIVITY	DESCRIPTION	REACH / NUMBER OF PARTICIPANTS
K-6 programming	Delivered educational programming to K-4 students at Logan Elementary; programming for 5–6 grade students delivered at Foley Library	260
Tabling at the Logan Block Party	Provided to-go bag with: Healthy snack (a smoothie) Recipe cards Body positivity stickers Handout detailing healthy food options in the Logan neighborhood	252
Educational placemats and handouts for the Logan Family Dinners	Provided handouts for both to-go and in-person dining (marketed towards adults): *Health Literacy: What It Is and Why It's Important* *Eating Disorders and Disordered Eating in Adolescents* *Nutritional Guidelines: Vitamins* *Intuitive Eating* *Healthy Body, Healthy Mind* Provided placemats for in person dining (marketed towards youth)	500 handouts and 100 placemats
Partner in supporting nutritious cooking classes for middle school students	Purchased cooking kits (mixing bowls, utensils, etc.) for students to take home	40
Build a toolkit for teachers	Toolkit built for elementary and middle school teachers on the topics of mental health, bullying, and body image. Included resources such as lesson planning and workshop curriculum.	From 8/22 - 12/22: 24 visits to the homepage; 293 visits to the Body Image resources page

To seek out other ways of connecting with the public, the project team scheduled multiple meetings with staff in the CCE. Through these conversations, the team was invited to have a table at the Logan Block Party, an annual event hosted by Gonzaga University, to be held in the spring of 2022. Though they were unable to give in-person presentations at the Logan Family Dinners, the project team was able to meet with members of the community through this new opportunity. Outreach funding from the grant allowed the team to build to-go bags that included ready-to-drink smoothies and printed materials, such as smoothie recipe cards and information about healthy food options in the Logan neighborhood; 252 bags were distributed at this event (see Table 2).

### Additional Opportunities: Making Connections

As they approached the end of the grant period, the project team met with administrators at Logan Elementary and the two local middle schools to discuss other ways grant funds could be utilized to support existing student health literacy efforts. In this meeting, the team learned that Gonzaga's Campus Kitchen was planning on providing nutrition literacy and cooking classes for 40 students during the Shaw Middle School summer programming; as this aligned with the project goals, the team partnered with Campus Kitchen to provide financial support for this program.

## DISCUSSION

As its primary goal, this project sought to promote health through two educational avenues: school programming at the local elementary school, and the distribution of health literacy materials to adult community members. The project team was successful in meeting this goal, delivering nutrition literacy education by providing educational sessions to approximately 260 students and distributing over 750 handouts to adults attending the Logan Block Party and the Logan Family Dinners. For the original grant writers (who came from a public health communication background), the focus of this grant was not necessarily about providing a measurable intervention for a problem, but about general public health communication and fostering community relationships. In this secondary goal the project was successful, as it further situated Gonzaga University as a potential partner for future community-based health projects, evidenced by the fact that the university has been asked to participate in another similar project.,

A major strength of this project implementation was the constant communication not only with external organizations, but also within the team's own institution. This project initially detailed only two avenues for providing health literacy education, but through conversations with both internal and external stakeholders, the project team was able to identify additional ways of reaching the community, such as tabling at the local Block Party. The author met with elementary and middle school administrators to discuss other ways the team could assist with health literacy initiatives, which led to partnering with the Campus Kitchen at Gonzaga on healthy cooking classes for middle school students.

However, the methods for how this project was accomplished are potentially problematic, as it was built on the assumption that this intervention was necessary. The aims and outcomes of this project were determined by educators and administrators from Gonzaga and the local elementary school, but the voices of community members were not consulted. The issue of low health literacy in students was not fully defined, nor was it evident that providing nutrition education to students and families would address a specific deficiency. While the Washington State Food Security Survey found that people in Spokane self-reported a decrease in healthy eating during the pandemic [22], there was no evidence that this was caused by low levels of health literacy, or that nutrition literacy would help remedy this problem. The survey also noted that during the pandemic, respondents reported seeing an increase in food prices and in using food assistance programs, suggesting that food insecurity was a key factor in the consumption of unhealthy foods [22]. Nutrition literacy was identified as an issue, but additional research is necessary to ascertain whether there is a true correlation between health literacy and healthy eating in this community.

Without consulting the community, the project team did not have the opportunity to take into consideration common reasons behind the food choices that people make. Food choices may be highly influenced by cultural norms, including social identity, ethnicity, religion, gender identity, and ethics [28]. Access to healthy food may hinge on a number of factors, including the financial privilege of purchasing more expensive healthy foods and having the time and physical ability to prepare meals. Additionally, access to healthier food options can be challenging for those who live in a “food swamp,” where there is an abundance of fast-food chains and convenience stores, but little access to affordable healthy food. These factors could have been identified by using community-based participatory research (CBPR), which engages residents in the process of identifying and planning community health interventions [32]; long-term processes such as CBPR can help to ensure that community health programs are conducted in full partnership with the community.

CBPR could have also helped mitigate the potential for educational elitism or saviorism. Colleges and universities have immense power within their local communities. Lopez and Romero outline two major issues with academics' civic engagement work: this work frames the community as having a deficiency, and it perpetuates savior mentalities amongst members of the academic institution, situating academic elites as intellectually superior [29]. The author was concerned about the possibility of perpetuating unexamined power and privilege in this project, and while methods for addressing community health literacy through an actively anti-racist framework are beginning to be discussed [30,31], scholarship in this area is still lacking.

The project also suffered from poor assessment planning and a lack of continuity from the initial grant writing to project implementation. As management of the project changed hands first from the School of Nursing & Human Physiology, then to Foley Library administrators, and finally to the author, a newly hired Health Sciences Librarian, vital information was lost in translation. The author had extensive project management experience as a former small library director, and while their leadership experience aided in navigating a project with a moving target, it did not change the fact they were new to the institution and did not yet fully understand the needs of the community or how the project originated.

### Opportunities for Academic Health Science Librarians

Despite the challenges of this case study, this project and the existing literature highlight various opportunities for academic libraries to be involved in health literacy outreach in their local communities. Pandemic-era projects have demonstrated innovative ways that academic health science librarians are promoting health literacy, such as planning and coordinating a healthcare conference for a large, multi-state health sciences university [33], or collaborating with medical school staff and students to provide public library health programming [10].

With these new opportunities, it is important that health science librarians approach this work with care and examine their roles in community health projects. While a primary goal in the field of public health is to improve health outcomes, public health initiatives have the potential to be incredibly harmful, especially for marginalized groups [34,35]. Viewing this historical context in combination with the power structures inherent in public health initiatives where an institution is situated as the experts within a community, there are many opportunities for missteps in community health programming. While risk and failure are commonplace and even expected, asking questions about potential harm to a community is vital for anyone conducting this work. A long-term, collaborative assessment process, such as the CBPR work done by Foell et al. [32, may be helpful in providing a collective framework for identifying ways to address community health needs. This process is time consuming, but successful health partnerships require time to build relationships, establish trust, and create programming that is long-lasting and sustainable.

For health science librarians, it is also important to identify library-specific skills and limitations and ask what the role of a library professional should look like in this context. For this particular project, the author had project management experience but did not have a public health background, a specialized knowledge of nutrition, or experience creating curriculum and teaching primary school children. Building a toolkit for educators was an excellent use of a health science librarian's specialized skillset, but other areas of this project could have been much better managed by individuals with experience and skills in those particular areas. The specialized knowledge of health science librarians in educating people about accessing information and assessing the reliability of online information is incredibly valuable in public health education, so finding opportunities to share those skills will better serve the communities we are working in. Health science librarians with project management skills also have the opportunity to help lead community health projects, but librarian project managers also need to ensure that they are not taking on work that would be better suited to another. This may mean being honest about limitations and seeking out expertise outside of their immediate circles. As new opportunities continue to arise with new funding opportunities, health science librarians can leverage their unique resources and knowledge to help build sustainable and mutually beneficial partnerships in the community.

## DATA AVAILABILITY STATEMENT

There are no data associated with this article. Requests for lesson plans or outreach materials can be made by contacting the author directly.

The associated toolkit, Addressing Mental Health in Children: A Health Literacy Toolkit for Educators, is built on LibGuides and is licensed under a Creative Commons Attribution 4.0 International License. Public access to the toolkit can be found at https://researchguides.gonzaga.edu/health-literacy-toolkit.

## AUTHOR CONTRIBUTIONS

Candise Branum: Conceptualization, data curation; investigation; methodology; project administration; supervision; writing—original draft; writing—review & editing.

## References

[1] Healthy People 2030. Health Literacy. [Internet]. U.S. Department of Health and Human Services, Office of Disease Prevention and Health Promotion. [cited 2023 Jun 29]. https://health.gov/healthypeople/priority-areas/social-determinants-health/literature-summaries/health-literacy.

[2] Whitney W, Keselman A, Humphreys B. Libraries and librarians: Key partners for progress in health literacy research and practice. Inf Serv Use. 2017 Apr;37(1):85–100. DOI: 10.3233/ISU-170821. PMC5724359 28972531

[3] Duhon L, Jameson J. Health information outreach: a survey of U.S. academic libraries, highlighting a midwestern university's experience. Health Inf Libr J. 2013 Jun;30(2):121–37. DOI: 10.1111/hir.12017. 23692453

[4] Barr-Walker J. Health literacy and libraries: a literature review. Ref Serv Rev. 2016 Jun 13;44(2):191–205. DOI: 10.1108/RSR-02-2016-0005.

[5] Zhang Y, Parry K. Health sciences and public librarians partnering to create a culture of health. J Consum Health Internet. 2018 Apr 3;22(2):102–11. DOI: 10.1080/15398285.2018.1434348.

[6] Ladd DL, Sobczak P, Tarver T. Providing health information to seniors: A program overview and reliable online senior health resources. J Consum Health Internet. 2019 Apr 3;23(2):113–22. DOI: 10.1080/15398285.2019.1608500.

[7] Howrey M, Marhenke C. Health aging resources for seniors and caregivers: An ehealth literacy library training partnership. Fla Libr. 2014 Jan 1;57(2):27–31.

[8] Aspinall EE, Beschnett A, Ellwood AF. Health literacy for older adults: Using evidence to build a model educational program. Med Ref Serv Q. 2012;31(3):302–14. DOI: 10.1080/02763869.2012.698174. 22853303

[9] Lou Strong M, Guillot L, Badeau J. Senior CHAT: A model for health literacy instruction. New Libr World. 2012 Jan 1;113(5/6):249–61. DOI: 10.1108/03074801211226337.

[10] Swanberg SM, Bulgarelli N, Jayakumar M, Look E, Shubitowski TB, Wedemeyer R, Yuen EW, Lucia VC. A health education outreach partnership between an academic medical library and public library: Lessons learned before and during a pandemic. J Med Libr Assoc. 2022 Apr 26;110(2):212–21. DOI: 10.5195/jmla.2022.1413. 35440901 PMC9014951

[11] Ottosen T, Mani NS, Fratta MN. Health information literacy awareness and capacity building: Present and future. IFLA J. 2019 Oct;45(3):207–15. DOI: 10.1177/0340035219857441.

[12] Olney CA, Warner DG, Reyna G, Wood FB, Siegel ER. Medlineplus and the challenge of low health literacy: Findings from the Colonias Project. J Med Libr Assoc. 2007 Jan;95(1):31–9. 17252064 PMC1773027

[13] Olney CA, Hansen L, Vickman A, Reibman S, Wood FB, Siegel E. Long-term outcomes of the ¡VIVA! Peer Tutor Project: use of MedlinePlus by former peer tutors and the adults they taught. J Med Libr Assoc JMLA. 2011 Oct;99(4):317–20. DOI: 10.3163/1536-5050.99.4.012. 22022228 PMC3193358

[14] Koos JA, Saragossi J, Stevens GA, Filosa S. A partnership between academic and public librarians: “What the Health” workshop series. J Med Libr Assoc JMLA. 2019 Apr;107(2):232–7. DOI: 10.5195/jmla.2019.564. 31019392 PMC6466498

[15] Engeszer RJ, Olmstadt W, Daley J, Norfolk M, Krekeler K, Rogers M, Colditz G, Anwuri VV, Morris S, Voorhees M, McDonald B, Bernstein J, Schoening P, Williams L. Evolution of an academic–public library partnership. J Med Libr Assoc JMLA. 2016 Jan;104(1):62–6. DOI: 10.3163/1536-5050.104.1.010. 26807055 PMC4722645

[16] Radick L. Improving health literacy, one public library at a time. Am Libr. 2015 Dec 11;46(11/12):48–53.

[17] Malone T, Clifton S. Using focus groups to evaluate a multiyear consumer health outreach collaboration. J Med Libr Assoc. 2021;109(4):575–82. DOI: 10.5195/jmla.2021.987. 34858086 PMC8608199

[18] Wu L, Blalock L, Cunningham K, Graysno M, Stephenson P. Wiring seniors to quality health information. J Consum Health Internet. 2006 Jul 24;10(2):11–24. DOI: 10.1300/J381v10n02_02.

[19] Kirkpatrick N, Dixson MA. An academic library utilization of research guides to disseminate consumer health resources. J Consum Health Internet. 2020 Oct 1;24(4):430–8. DOI: 10.1080/15398285.2020.1827898.

[20] Kershner J. Zag history 101. The Spokesman-Review [Internet]. 2007 Jul 8 [cited 2023 Oct 17]; https://www.spokesman.com/stories/2007/jul/08/zag-history-101/.

[21] U.S. Census Bureau. American Community Survey, 2019 ACS 1-Year estimates, Table DP05 [dataset]. [2019; cited 2023 Jan 3]. https://data.census.gov/table?q=Spokane+County,+Washington&tid=ACSDP1Y2019.DP05.

[22] Drewnowski A, Otten JJ, Lewis LR, Collier SM, Sivaramakrishnan B, Rose CM, Ismach A, Nguyen E, Buszkiewicz J. Food security and food access amid COVID-19 in Spokane County households, Research Brief 10 (Spokane County) [Internet]. 2021 Jul. (Washington State Food Security Survey). https://nutr.uw.edu/cphn/wafood/brief-10.

[23] Christoph MJ, Loth KA, Eisenberg ME, Haynos AF, Larson N, Neumark-Sztainer D. Nutrition facts use in relation to eating behaviors and healthy and unhealthy weight control behaviors. J Nutr Educ Behav. 2018 Mar;50(3):267–274.e1. DOI: 10.1016/j.jneb.2017.11.001. 29276019 PMC5845784

[24] O'Dea JA. Developing positive approaches to nutrition education and the prevention of child and adolescent obesity: First, do no harm. In: Childhood Obesity Prevention: International Research, Controversies, and Interventions. Oxford University Press; 2010.

[25] MyPlate [Internet]. [cited 2023 Aug 31]. Available from: https://www.myplate.gov/.

[26] Fernandez ML, Raheem D, Ramos F, Carrascosa C, Saraiva A, Raposo A. Highlights of current dietary guidelines in five continents. Int J Environ Res Public Health. 2021 Mar 10;18(6):2814. DOI: 10.3390/ijerph18062814. 33802065 PMC8001548

[27] Health Education Curriculum Analysis Tool (HECAT) [Internet]. 2023 [cited 2023 Aug 31]. Available from: https://www.cdc.gov/healthyyouth/hecat/index.htm.

[28] Gerber S, Folta SC. You Are What You Eat… But Do You Eat What You Are? The Role of Identity in Eating Behaviors—A Scoping Review. Nutrients. 2022 Jan;14(17):3456. DOI: 10.3390/nu14173456. 36079713 PMC9458161

[29] Lopez E, Romero J. Integrating civic engagement and ethnic studies in campus outreach: the case of Aquetza. J Coll Character. 2017 Oct 2;18(4):296–305. DOI: 10.1080/2194587X.2017.1371044.

[30] Agarwal A, Crawford N, Nguyen V, Walker A. The White Savior Industrial Complex in global health [Internet]. BMJ Global Health blog. 2020 [cited 2022 Dec 25]. https://blogs.bmj.com/bmjgh/2020/03/11/the-white-savior-industrial-complex-in-global-health.

[31] Heal & racial justice [Internet]. HEAL Initiative. [cited 2022 Dec 25]. https://healinitiative.org/racial-justice/.

[32] Foell A, Purnell JQ, Barth R, Witthaus M, Murphy-Watson T, Martinez S, Foley M. Resident-Led Neighborhood Development to Support Health: Identifying Strategies Using CBPR. Am J Community Psychol. 2020 Dec;66(3–4):404–16. DOI: 10.1002/ajcp.12441. 33161586

[33] Alcorn KS, McCord SK, Seed SM, Gravel T, Morrill AM. The veteran-centered care conferences: Interprofessional education and community involvement facilitated by the health sciences librarian. J Med Libr Assoc. 2022 Dec 8;110(3):365–71. DOI: 10.5195/jmla.2022.1491. 36589306 PMC9782503

[34] Spector-Bagdady K, Lombardo PA. U.S. Public Health Service STD experiments in Guatemala (1946-1948) and their aftermath. Ethics Hum Res. 2019 Mar;41(2):29–34. DOI: 10.1002/eahr.500010. 30895754

[35] Tobin MJ. Fiftieth anniversary of uncovering the Tuskegee Syphilis Study: The story and timeless lessons. Am J Respir Crit Care Med. 2022 May 15;205(10):1145–58. DOI: 10.1164/rccm.202201-0136SO. 35500908 PMC9872801

